# Planarian ‘kidneys’ go with the flow

**DOI:** 10.7554/eLife.09353

**Published:** 2015-07-22

**Authors:** Melanie Issigonis, Phillip A Newmark

**Affiliations:** Department of Cell and Developmental Biology, Howard Hughes Medical Institute, University of Illinois at Urbana-Champaign, Urbana, United States; Department of Cell and Developmental Biology, Howard Hughes Medical Institute, University of Illinois at Urbana-Champaign, Urbana, United Statespnewmark@life.illinois.edu

**Keywords:** planarian, excretory system, cystic kidney disease, other

## Abstract

Flatworms have organs called protonephridia that could be used as a model system for the study of kidney disease.

**Related research article** Thi-Kim Vu H, Rink JC, McKinney SA, McClain M, Lakshmanaperumal N, Alexander R, Sánchez Alvarado A. 2015. Stem cells and fluid flow drive cyst formation in an invertebrate excretory organ. *eLife* **4**:e07405. doi: 10.7554/eLife.07405

**Image** Planarian protonephridia could help us understand the causes of kidney diseases in mammals

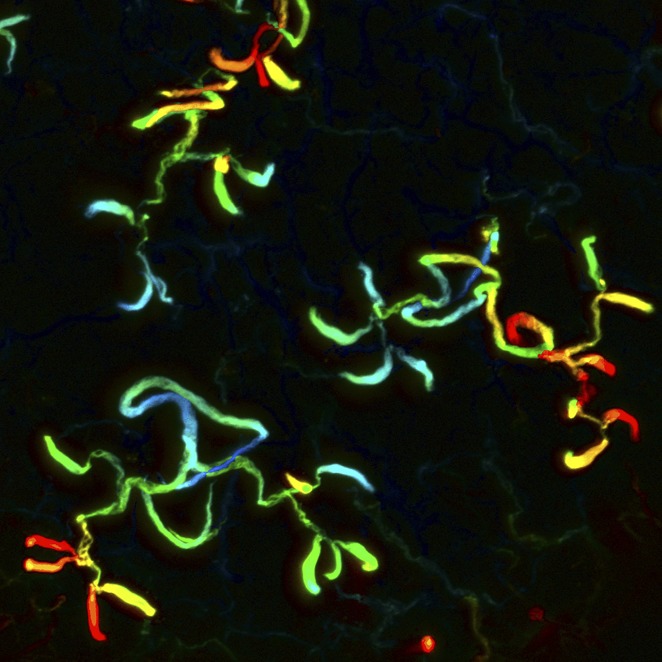

The human kidney filters an astonishing 170 liters of blood every day to remove metabolic wastes from our bodies. Our kidneys also balance the body's fluids and release hormones that control blood pressure. Any disruption to normal kidney function can lead to serious health issues and, in some cases, death. Cystic kidney diseases affect millions of people worldwide and comprise a wide range of inherited and acquired diseases that are all caused by the formation of fluid-filled cysts in the kidney (Bisceglia et al., 2006). Many of the mutations that cause cystic kidney diseases affect genes that are important for the structure or function of cilia—the tiny hair-like structures that perform a number of important roles in the kidney (Yoder et al., 2002; Mollet et al., 2005; Fliegauf et al., 2006). This supports the notion that the same molecular mechanisms drive cyst formation in a range of cystic kidney diseases.

The fruit fly *D. melanogaster* and the roundworm *C. elegans* have been essential model organisms for studying development and disease, in part because it is relatively easy to create mutants that allow the functions of individual genes to be investigated. However, the excretory cells in these two species do not have cilia, which has limited their use as models for kidney disease. Now, in *eLife*, Alejandro Sánchez Alvarado and colleagues—including Hanh Vu as first author—report that structures called protonephridia in planarian flatworms could be used as an invertebrate model of the mammalian kidney (Thi-Kim Vu et al., 2015).

Our kidneys are made up of about a million filtering units called nephrons, which are divided into two subunits: a glomerulus and a tubule. The glomerulus acts as a highly selective filter that allows water and small solutes (for example, urea) to pass out of the bloodstream and into the tubule, while preventing circulating cells and large proteins from doing so. The tubule then redirects important minerals and molecules back into the bloodstream, with different segments of the tubule able to reabsorb different substances (Scott and Quaggin, 2015; Figure 1). Waste substances are not reabsorbed and continue down the tubule to be excreted in the urine.

**Figure 1.**
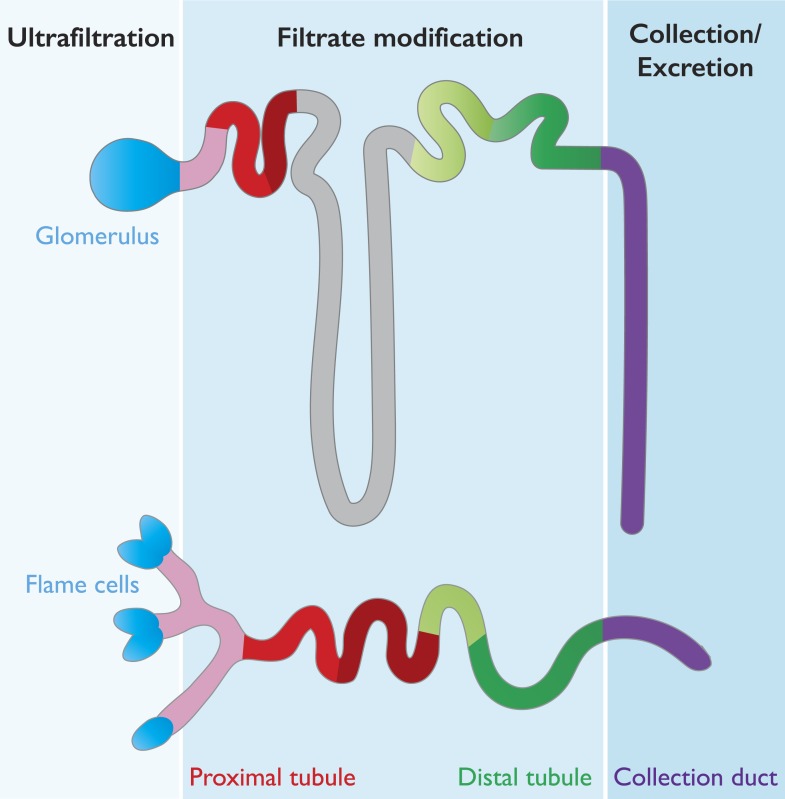
Many features are conserved between the mammalian nephron and planarian protonephridia. Cartoon depiction of the structural and physiological components in the mammalian nephron (top), and the planarian protonephridia (bottom). Both the nephron and protonephridia are made up of several different subregions. Water and small molecules are filtered out of the bloodstream by the glomerulus in mammals and flame cells in planarians (blue). Useful substances are then reabsorbed into the bloodstream as they travel along the tubule (pink, red, gray and green; each color represents an area where different molecules are taken back up into the body). Finally, water and metabolic wastes pass into the collection duct (purple) and are excreted. Vu et al. suggest that these similarities make the planarian protonephridia a good model of the mammalian nephron.

The planarian excretory system is made up of protonephridia, which are branched organs that are widely distributed throughout the body. Protonephridia consist of ciliated ‘flame cells’ connected to long tubules (some of which are ciliated) that end at the surface of the planarian (Rink et al., 2011; Scimone et al., 2011; Figure 1).

Although the anatomy of planarian protonephridia is quite different from that of the mammalian kidney, Vu et al.—who are based at the Stowers Institute for Medical Research and the Max Planck Institute of Molecular Cell Biology and Genetics—show that both have remarkably similar roles. To investigate whether planarian protonephridia serve as ultrafiltration units, Vu et al. injected fluorescent tracers with different molecular weights into the animal and found that protonephridia preferentially took up the tracers with lower molecular weights. This observation confirmed that, similar to the vertebrate kidney, protonephridia act as filters.

To characterize the protonephridia in detail, Vu et al. identified all the genes in the planarian genome that encode the solute carrier (slc) transporter proteins that move molecules into and out of kidney cells. They identified a total of roughly 300 slc proteins. Mapping the expression patterns of these genes using a technique called fluorescent in situ hybridization revealed that about 15% of the transporter genes were expressed in the protonephridia. Vu et al. then defined at least six separate segments of the protonephridia, and found that solute carriers with shared transport activities were expressed in similar segments.

Strikingly, the sequence of these segments—and the tasks they perform—was similar in both the planarian protonephridia and the vertebrate nephron. For example, in the vertebrate nephron the distal tubule regulates pH levels by reabsorbing bicarbonates from the filtrate and secreting protons into the urine. Similarly, planarian protonephridia express bicarbonate and proton transporters in the equivalent segments of their distal tubule (Figure 1). Moreover, RNA interference knockdown of a bicarbonate transporter led to a corresponding increase in the intercellular pH of the planarian.

The structural, molecular and physiological conservation between planarian protonephridia and the mammalian nephron even extends to common pathologies. In humans, mutations in the nephrin family of proteins disrupt the filtration capabilities of the glomerulus and lead to nephrotic syndrome (Kestila et al., 1998; Donoviel et al., 2001). Vu et al. found that planarian equivalents of these proteins—*NPHS1-6* and *NEPH-3*—were expressed in flame cells; knockdown of these genes resulted in edema, which is a sign of excretory system problems. These results suggest that flame cells are the planarian version of the vertebrate glomerulus.

Vu et al. also identified planarian equivalents of human genes that cause cystic kidney diseases when mutated. Mutations in some of these genes result in nephronophthisis, which is the most common genetic cause of childhood kidney failure (Salomon et al., 2009). Moreover, the knockdown of these genes in planarians (nephrocystin genes, to be precise) led to structural defects in protonephridial tubules and the development of cyst-like structures.

The remarkably similar pathologies in humans and planarians even extend to a common underlying mechanism. In humans, renal cysts result from uncontrolled cell proliferation. Similarly, the number of protonephridial progenitor cells increased in nephrocystin knockdown animals and led to edema. Furthermore, increasing or decreasing global cell proliferation led to a corresponding exacerbation or reduction in cyst size and edema. Human cystic kidney diseases are disorders that affect the cilia; therefore, Vu et al. investigated the effect of knocking down genes required for cilia structure or function in planarians. As well as problems with cilia-driven locomotion, the animals displayed increased protonephridial progenitor cell proliferation, which led to cyst development and edema. Therefore, cilia are important for sensing and generating fluid flow in planarians.

The work of Vu et al. uncovers the extraordinary conservation between planarian protonephridia and the mammalian kidney. These studies suggest a common evolutionary origin of animal excretory systems and firmly establish planarians as an excellent model in which to study human kidney function and disease. There is still much that is unknown in this field, including the mechanisms that connect flow sensing and progenitor cell proliferation, and the identity of the over-proliferative cells that lead to renal cysts.

Given the relative ease with which the roles of genes can be investigated in planarians, future work on these animals may help to resolve many of these outstanding questions.
